# Anemia in Heart Failure: Diagnostic Insights and Management Patterns Across Ejection Fraction Phenotypes

**DOI:** 10.3390/diagnostics15162079

**Published:** 2025-08-19

**Authors:** Otilia Țica, Ovidiu Țica

**Affiliations:** 1 Cardiology Clinic, Emergency County Clinical Hospital of Bihor, 410165 Oradea, Romania; 2 Department of Morphological Disciplines, Faculty of Medicine and Pharmacy, University of Oradea, 410073 Oradea, Romania; 3 Pathology Department, Emergency County Clinical Hospital of Bihor, 410165 Oradea, Romania

**Keywords:** anemia, heart failure, HFpEF, HFrEF, HFmrEF, phenotype-tailored medicine

## Abstract

**Background:** Anemia is a common comorbidity in heart failure (HF) and has been associated with adverse clinical consequences. This retrospective, descriptive cohort study examined phenotype-specific differences in anemia severity, clinical presentation, comorbid burden, and in-hospital management across HF subtypes classified by left ventricular ejection fraction (LVEF). **Methods:** We retrospectively analyzed 443 adult patients hospitalized with concurrent HF and anemia from January 2022 to December 2024. Patients were stratified by LVEF into HFrEF (<40%), HFmrEF (40–49%), and HFpEF (≥50%). All patients included met WHO criteria for anemia. Demographic, clinical, paraclinical, and therapeutic data were extracted, and descriptive statistical methods were used to evaluate intergroup differences. No formal time-to-event analyses (e.g., Kaplan–Meier curves) were performed; instead, exploratory cumulative readmission analyses using fixed follow-up windows were conducted. In-hospital mortality was recorded and stratified by HF phenotype. **Results**: The cohort comprised 213 (48.0%) HFrEF, 118 (26.6%) HFmrEF, and 112 (25.3%) HFpEF patients. The distribution of anemia severity, management strategies, and comorbidity profiles varied significantly across phenotypes. Severe anemia predominated in the HFmrEF cohort (54.2%), whereas mild anemia was most common in HFpEF (52.1%) and HFrEF (52.1%). Mean hemoglobin concentrations were 8.39 ± 1.79 g/dL (HFmrEF), 9.07 ± 2.47 g/dL (HFpEF), and 8.62 ± 1.94 g/dL (HFrEF). Rates of atrial fibrillation (48.2% in HFpEF), hypertensive ECG changes (63.4% in HFpEF), and ischemic-lesion patterns (>50% in HFrEF) differed by cohort. Echocardiographically, grade III mitral regurgitation and severe pulmonary hypertension each affected 25.4% of HFmrEF patients, whereas HFpEF patients most often exhibited grade II mitral regurgitation (42.9%) and moderate pulmonary hypertension (42.9%). HFrEF patients had severe pulmonary hypertension. Intravenous (IV) iron was the primary treatment modality, with highest utilization in HFmrEF. IV iron use ranged from 69.9% (HFrEF) to 84.8% (HFmrEF), with transfusion rates of 5.6% (HFrEF)–16.1% (HFpEF). Comorbid burdens differed by phenotype: HFrEF was associated with structural heart disease, HFmrEF with vascular and hepatic pathology, and HFpEF with metabolic and degenerative comorbidities. Discharge pharmacotherapy reflected phenotype-specific treatment patterns. **Conclusions**: This real-world descriptive analysis highlights substantial variation in anemia burden and management across the HF spectrum. While limited to descriptive findings, our analysis highlights the heterogeneity of anemia in HF and describes observed associations across phenotypes, without implying causality. These findings should be interpreted as hypothesis-generating. These findings are observational, exploratory, and cannot establish a causal relationship between intravenous iron use and survival.

## 1. Introduction

Heart failure (HF) remains a leading cause of morbidity and mortality worldwide, with an estimated 64 million individuals affected globally [1]. Despite advancements in guideline-directed medical therapy—including renin–angiotensin–aldosterone system inhibitors, beta-blockers, mineralocorticoid receptor antagonists, and sodium–glucose cotransporter 2 (SGLT2) inhibitors—long-term outcomes remain suboptimal [2,3]. Comorbid conditions, particularly anemia, further exacerbate HF progression by reducing oxygen delivery, increasing myocardial workload, and promoting neurohormonal activation [4,5,6].

Anemia complicates HF in approximately 20–50% of patients, with prevalence varying by HF phenotype, patient selection, and diagnostic criteria [7]. Its multifactorial pathophysiology includes chronic inflammation (mediated by elevated interleukin-6 and hepcidin), renal insufficiency reducing erythropoietin production, hemodilution secondary to fluid overload, nutritional deficiencies (iron, vitamin B12, folate), and adverse effects of certain HF pharmacotherapies on erythropoiesis [8,9]. Observational studies have consistently shown that anemia in HF correlates with reduced exercise tolerance, poorer quality of life, increased hospitalization rates, and higher all-cause mortality [10,11].

Clinical trials over the last decade have largely focused on intravenous (IV) iron replacement in HF with reduced ejection fraction (HFrEF). The FAIR-HF [4] and CONFIRM-HF [12] trials demonstrated that IV ferric carboxymaltose significantly improves functional capacity (6 min walk distance), symptoms (NYHA class), and quality of life among HFrEF patients with iron deficiency, irrespective of baseline anemia status [13]. AFFIRM-AHF [14] extended these findings by showing that IV iron administered early after acute decompensation reduced HF rehospitalizations at 52 weeks. More recently, the IRONMAN trial [15] confirmed that routine administration of IV ferric derisomaltose in HFrEF patients with iron deficiency produced sustained improvements in functional status and reduced composite HF hospitalizations and cardiovascular death [16]. Meta-analyses have consolidated these data, reporting a 25% relative risk reduction in HF hospitalizations with IV iron among HFrEF patients [13]. Although anemia prevalence in HF has been previously described, few studies have focused on inpatient populations stratified by LVEF in resource-limited settings with detailed therapy data.

However, evidence in heart failure with preserved ejection fraction (HFpEF) and heart failure with mid-range ejection fraction (HFmrEF) remains sparse. HFpEF comprises nearly half of all HF cases and is characterized by diastolic dysfunction, endothelial dysfunction, and comorbidity-driven systemic inflammation [2]. Anemia in HFpEF is associated with worse functional outcomes and increased mortality, but randomized data on IV iron in this subgroup are limited to small, single-center pilot studies, which demonstrated improved exercise capacity but lacked statistical power [17]. Understanding how anemia presents and responds to therapy in HFmrEF is critical but under-investigated.

Given these gaps, this retrospective analysis of adult patients discharged with concurrent anemia and HF aimed to: (1) characterize anemia severity, clinical presentations, and paraclinical findings (laboratory, electrocardiography, echocardiography) across HFrEF, HFmrEF, and HFpEF phenotypes; (2) document real-world anemia management strategies—including IV iron formulations, oral iron, and blood transfusions—and associated adverse events; (3) describe the comorbidity burden and discharge pharmacotherapy; and (4) identify potential phenotype-specific differences to inform tailored anemia management in HF. Given the retrospective design and lack of systematic follow-up, our study did not evaluate long-term outcomes but rather aimed to generate hypotheses for future prospective investigations. This study uniquely focuses on hospitalized Eastern European patients with detailed characterization of anemia severity, therapy modality, and in-hospital mortality across HF phenotypes—a population underrepresented in prior registry data.

## 2. Methods

### 2.1. Study Design and Population

A retrospective, observational, and descriptive study was conducted at the Cardiology Clinic, Emergency County Clinical Hospital of Bihor. We identified all adult patients (aged >18 years) discharged between January 2022 and December 2024 with clinical diagnoses of both HF and anemia. HF was diagnosed according to the actual ESC HF Guidelines by clinical criteria, encompassing typical symptoms, physical examination findings, and evidence of structural or functional cardiac impairment, and patients were subsequently stratified by left ventricular ejection fraction into HFrEF (<40%), HFmrEF (40–49%), and HFpEF (≥50%). All patients included met WHO criteria for anemia. The primary analysis cohort comprised only anemic patients; however, for secondary comparisons, a comparator cohort of HF patients without anemia was also identified. Patients transferred into the Cardiology Clinic from other departments or clinics were included if they met the above criteria on discharge. Medical records were reviewed to extract demographic data, comorbidities, presenting symptoms, laboratory results, electrocardiography (ECG), transthoracic echocardiography, anemia treatments, and discharge medications.

Patients were eligible for inclusion if they were admitted to the Emergency County Clinical Hospital of Bihor between January 2022 and December 2024 and discharged with concurrent clinical diagnoses of heart failure and anemia. All participants were aged over 18 years and had undergone at least one comprehensive laboratory assessment, including a complete blood count, ferritin quantification, and serum iron measurement, before discharge. To capture the full spectrum of cardiology referrals, we also included patients who were initially admitted to other departments or external clinics and subsequently transferred into our clinic. Importantly, cases were retained in the analysis regardless of in-hospital mortality during the index admission, provided they met the above criteria.

Patients were excluded if they did not have concurrent diagnoses of heart failure and anemia or lacked documentation of at least one complete laboratory panel, including a complete blood count and iron indices, before discharge. Cases in which anemia was attributable to a known secondary cause (such as malignancy) or an acute hemorrhagic event were also excluded. Patients readmitted to our clinic during the study period, those who developed an acute non-cardiac condition necessitating transfer to another specialty or more severe non-cardiac decompensation, and any patients transferred from our clinic to an external facility for any reason were likewise excluded.

In addition, a comparator cohort of HF patients without anemia, admitted during the same period and meeting identical HF diagnostic criteria, was identified. Anemia status was defined according to WHO criteria, and all patients in the comparator cohort met the definition of “no anemia”. This allowed for direct comparisons between HF patients with and without anemia across phenotypes.

### 2.2. Data Collection

Data were extracted through a comprehensive review of both electronic and paper medical records. Demographic information, including age, sex, and area of residence (urban versus rural), was recorded for each patient. Clinical presentation at admission was documented by noting the presence or absence of key signs and symptoms of cardiac decompensation and exercise intolerance as well as anemia-related manifestations (chest pain, anorexia, palpitations, presyncope, nausea, diaphoresis, headache, dizziness, and balance disturbances). Laboratory parameters were obtained from admission and routine pre-discharge assays and included hemoglobin, hematocrit, ferritin, serum iron, and transferrin when available. Across all heart failure (HF) phenotypes, complete blood count (CBC) was available and formed the basis for anemia stratification, while iron parameters (ferritin, serum iron, transferrin saturation) were available. Anemia severity among patients with heart failure (HF) was categorized according to World Health Organization (WHO) criteria as mild (Hb 10–12 g/dL in women or 10–13 g/dL in men), moderate (Hb 8 to <10 g/dL), or severe (Hb < 8 g/dL). All patients underwent standard 12-lead resting electrocardiography (ECG) upon admission. Phenotype-specific patterns were observed, reflective of structural and rhythm-based variations in HF subtypes. Transthoracic echocardiography was systematically performed in all patients during hospitalization. In addition to left ventricular ejection fraction (LVEF)-based classification, key structural and valvular abnormalities were recorded. Details of anemia management during hospitalization were also collected (intravenous iron or oral iron supplements, or the administration of blood transfusions). Finally, comorbid conditions and discharge pharmacotherapy were extracted to better characterize each patient’s overall treatment profile. Although long-term outcomes were not available, in-hospital mortality was captured and descriptively analyzed across heart failure phenotypes.

Decisions regarding the use of intravenous or oral iron therapy were made by the treating cardiologist according to clinical judgment, availability of specific preparations, and patient comorbidities. No standardized institutional protocol for iron supplementation was in place during the study period, and the rationale for selecting IV versus oral iron was not uniformly documented in the medical records.

### 2.3. Statistics

Continuous variables are presented as mean ± standard deviation (SD) and categorical variables as frequency (percentage). Bivariate comparisons between cohorts were performed using chi-square tests (for categorical data) or one-way analysis of variance (ANOVA) (for continuous data). Pairwise associations between variables were tested to determine statistical significance. To identify independent predictors of cohort membership and potential confounders, multinomial logistic regression was employed. Where variables demonstrated a skewed distribution (for example, hemoglobin, transferrin, power, and risk scores), these were normalized for statistical analysis by taking the natural logarithm. Kruskal–Wallis testing was used for non-normally distributed variables with exploratory adjusted logistic regression models employed for in-hospital mortality. Group comparisons across the three EF phenotypes were performed using one-way ANOVA for normally distributed continuous variables, the Kruskal–Wallis test for non-normally distributed continuous variables, and the chi-square test (or Fisher’s exact test when appropriate) for categorical variables. Normality was assessed using the Shapiro–Wilk test. A two-sided *p*-value < 0.05 was considered statistically significant. We performed exploratory adjusted analyses for the association between intravenous iron and in-hospital mortality using ridge-penalized multivariable logistic regression. Covariates included age, sex, residence, NT-proBNP, oral iron, transfusion, atrial fibrillation, comorbidities, and anemia severity. We also estimated propensity scores and fit stabilized inverse-probability weighted models. Given the observational design and treatment imbalance, results are interpreted as associations, not causal effects. While no formal survival analysis was performed due to the absence of exact event dates, exploratory cumulative readmission analyses using fixed post-discharge intervals were conducted (Section 2.4 and Section 2.5). No multivariable models were employed for long-term outcomes or anemia severity predictors; however, adjusted logistic and multinomial regression models were employed for in-hospital mortality and HF phenotype predictors, respectively. Results are presented as means ± standard deviation or percentages, with comparisons performed using appropriate bivariate statistics. Variables included in the multinomial logistic regression model were selected based on clinical relevance, prior studies, and statistically significant associations in bivariate analyses (*p* < 0.10). To assess multicollinearity, variance inflation factors (VIFs) were calculated, and all included predictors had VIF values < 2, indicating acceptable levels. Model fit was evaluated using McFadden’s pseudo-R^2^ and the likelihood ratio test, both of which demonstrated acceptable explanatory power and model adequacy. To account for potential confounding, multinomial logistic regression was employed to evaluate independent associations with HF phenotype. Multinomial logistic regression was used to identify independent predictors of HF phenotype, including anemia severity, atrial fibrillation, and residential setting.

A two-tailed *p*-value of <0.05 was considered statistically significant. Analyses used complete case data as the amount of missing data was small (no imputation performed). Statistical analysis was performed with Stata (version 17, StataCorp LP, College Station, TX, USA).

### 2.4. Exploratory Cumulative Readmission Analysis

We lacked exact post-discharge dates of readmission or death; therefore, we constructed stepwise cumulative readmission curves using fixed windows recorded in the dataset (≤30, ≤60, ≤90, and ≤180 days). Patients who died in hospital were excluded. For each window, we calculated the cumulative proportion with ≥1 readmission by that timepoint and compared groups using chi-square tests (Fisher’s exact when applicable). These plots are descriptive and do not represent Kaplan–Meier estimates.

### 2.5. Exploratory Cumulative Readmission Analyses (Subgroups)

Because exact post-discharge dates were unavailable, we constructed stepwise cumulative readmission curves using fixed windows recorded in the dataset (≤30, ≤60, ≤90, and ≤180 days). Patients who died in hospital were excluded. We compared subgroups defined by anemia severity (moderate vs. severe) and by treatment modality (IV iron (any IV) vs. oral iron only vs. no iron) using chi-square tests (Fisher’s exact when appropriate) at each time point. These plots are descriptive and do not represent Kaplan–Meier estimates; log-rank tests were not applied.

All statistical results for these subgroup analyses, including *p*-values and sample sizes, are presented directly in the Results section; no Supplementary tables were created.

## 3. Results

### 3.1. Patient Distribution and Demographics

Out of 462 patients who initially met the inclusion criteria, 19 were excluded due to incomplete data, yielding a final analytic cohort of 443 individuals. HF patients were stratified into three groups: 213 patients (48.0%) with HFrEF (LVEF < 40%), 118 patients (26.6%) with HFmrEF (LVEF 40–49%), and 112 patients (25.3%) with HFpEF (LVEF ≥ 50%).

In the HFmrEF cohort (*n* = 118), patient ages ranged from 35 to 94 years (mean ± SD, 74.9 ± 10.5 years), with a near-equal sex distribution (58 males, 49.2%; 60 females, 50.8%) and residence patterns of 47.5% urban versus 52.5% rural. The HFpEF group (*n* = 112) encompassed patients aged 32 to 94 years (mean ± SD, 71.8 ± 10.5 years), of whom 47 were male (40.2%) and 71 were female (59.8%), with 57.1% living in urban areas and 42.9% in rural settings.

The HFrEF cohort (*n* = 213) ranged in age from 34 to 93 years (mean ± SD, 77.2 ± 10.4 years), included 77 males (44.6%) and 96 females (55.4%), and comprised 41.8% urban and 58.2% rural residents.

In adjusted models, factors such as severe anemia, presence of atrial fibrillation, and rural residence were independently associated with the HFmrEF phenotype.

### 3.2. Anemia Severity

**Figure 1:**
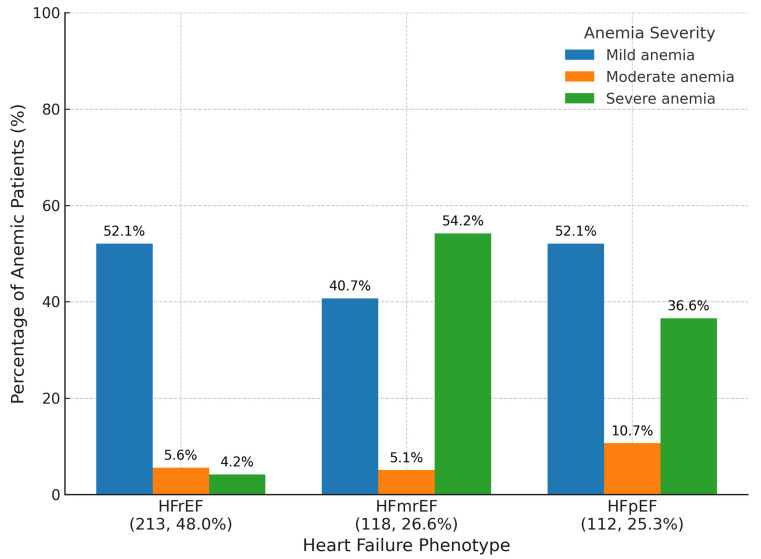
Anemia severity by heart failure phenotype. Clustered bar chart showing the percentage of patients with mild, moderate, and severe anemia across three heart failure groups (HFrEF, HFmrEF, HFpEF). Bars are color-coded by anemia severity, with exact percentages annotated and cohort sizes noted beneath each phenotype. HFmrEF, heart failure with mildly reduced ejection fraction; HFpEF, heart failure with preserved ejection fraction; HFrEF, heart failure with reduced ejection fraction.

Distribution patterns varied substantially across HF phenotypes (HFrEF, HFmrEF, and HFpEF), highlighting phenotype-specific differences in hematologic burden as seen in Figure 1.

In the HFmrEF cohort, severe anemia was strikingly predominant, affecting 54.2% of patients, followed by mild anemia in 40.7% and moderate anemia in only 5.1%. This unique pattern suggests a higher burden of systemic illness or inflammatory state within this group, consistent with prior reports linking HFmrEF to adverse prognostic features.

Conversely, among HFpEF patients, mild anemia was the most frequent (52.1%), with moderate and severe forms affecting 10.7% and 36.6% of the cohort, respectively. This distribution may reflect the chronicity of comorbid conditions, such as renal dysfunction and frailty, common in older adults with preserved ejection fraction.

For HFrEF patients, the pattern was similarly dominated by mild anemia (52.1%), with moderate anemia present in 5.6% and severe anemia in 4.2% of patients. This relative predominance of mild forms may reflect established treatment pathways and earlier recognition in this phenotype.

### 3.3. Presenting Complaints and Clinical Profiles

**Figure 2:**
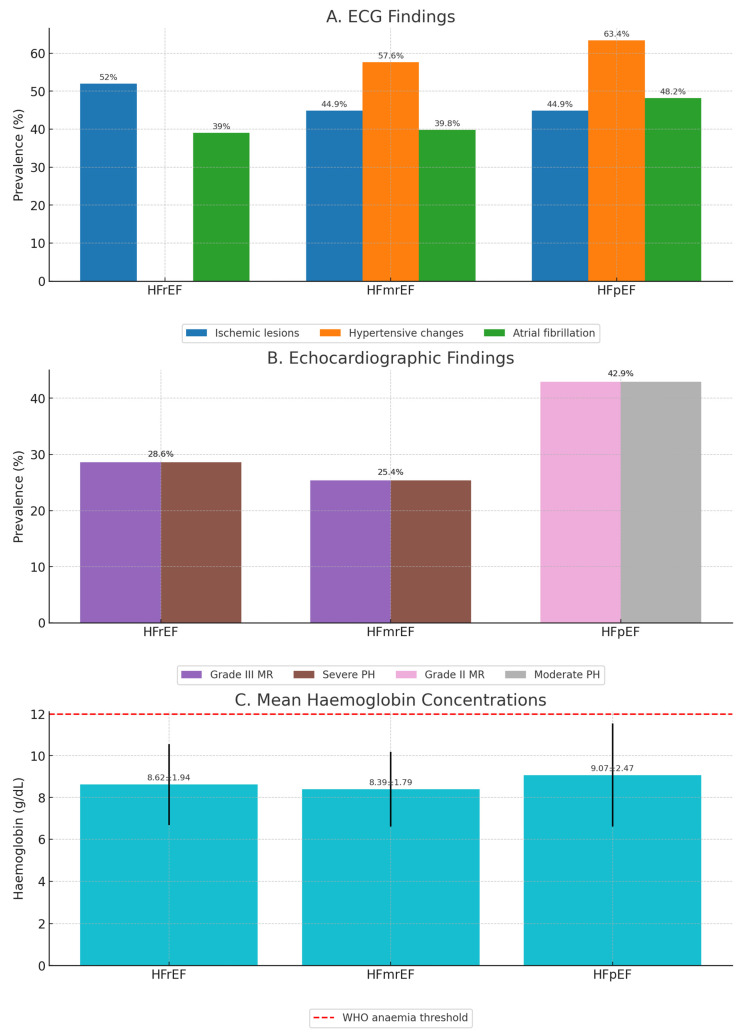
Paraclinical findings across heart failure phenotypes. (**A**) Electrocardiographic (ECG) abnormalities: prevalence (%) of ischemic lesions, hypertensive changes, and atrial fibrillation (AF) in heart failure with reduced ejection fraction (HFrEF), mildly reduced ejection fraction (HFmrEF), and preserved ejection fraction (HFpEF). Bars are color-coded by abnormality type, with exact percentages displayed above each bar. (**B**) Echocardiographic findings: prevalence (%) of valvular regurgitation (grade II and grade III mitral regurgitation (MR)) and pulmonary hypertension (PH; moderate and severe) across phenotypes. Bars are color-coded by finding, with exact percentages displayed above each bar. (**C**) Mean hemoglobin concentrations: mean ± standard deviation (g/dL) in each phenotype, with error bars representing standard deviation. The dashed red line marks the World Health Organization (WHO) anemia threshold for reference. Abbreviations: ECG, electrocardiography; HFpEF, heart failure with preserved ejection fraction; HFmrEF, heart failure with mildly reduced ejection fraction; HFrEF, heart failure with reduced ejection fraction; MR, mitral regurgitation; PH, pulmonary hypertension; WHO, World Health Organization.

In the context of HF and coexistent anemia, clinical presentation at admission revealed distinct profiles across ejection fraction phenotypes, reflecting variations in hemodynamic burden and anemia-associated symptomatology. These findings offer clinically meaningful insights into diagnostic patterns and symptom clustering relevant to phenotype-specific management as seen in Figure 2.

In the HFmrEF cohort, symptoms of both cardiac decompensation and hematologic origin were prominent. Dyspnoea (50.0%) and asthenia (64.4%) were leading complaints, while reduced exercise tolerance (39.8%) and fatigability (30.5%) further characterized decompensation. Clinical signs such as peripheral oedema and crepitant rales were noted in 29.7% of patients, highlighting fluid overload. Notably, anemia-specific symptoms were highly prevalent: chest pain (39.8%), anorexia (25.4%), palpitations (14.4%), and presyncope (10.2%) frequently coexisted with classic HF signs, suggesting a compounded symptom burden that may complicate initial clinical evaluation.

In contrast, patients with HFpEF displayed more pronounced systemic and anemia-related features. Dyspnoea (75.0%) and fatigability (58.9%) remained predominant, accompanied by asthenia (48.2%), peripheral oedema (46.4%), and rales (16.1%). Anemia-related complaints were particularly pronounced in this phenotype: dizziness (42.0%), anorexia (42.9%), chest pain (37.5%), and diaphoresis (31.3%) were frequently documented. Furthermore, 16.1% reported nausea and presyncope, while balance disturbances affected over 10%, a constellation of findings suggestive of systemic hypoperfusion and frailty.

Patients with HFrEF exhibited a presentation dominated by signs of advanced cardiac decompensation. Dyspnoea (72.3%), fatigability (69.9%), and asthenia (64.8%) were highly prevalent. Peripheral oedema (65.3%), crepitant rales (57.8%), and reduced exercise tolerance (53.1%) defined a classic congestive profile. Additionally, orthopnoea (22.5%), cardiogenic shock (13.6%), and oliguria (10.8%) were recorded in more severe cases. Despite this, anemia-related symptoms were also considerable: chest discomfort (32.9%), diaphoresis (31.5%), palpitations (29.6%), and headache (19.7%) co-occurred, underscoring the multifactorial nature of their clinical status. Dizziness (16.9%), anorexia (15.0%), presyncope (10.8%), nausea (10.3%), and balance issues rounded out the clinical spectrum.

### 3.4. Paraclinical Investigations

Figure 2 summarizes phenotype-specific differences in ECG, echocardiographic, and laboratory parameters.

ECG findings (Figure 2A): Ischemic lesion patterns were most frequent in HFrEF (>50%), followed by HFmrEF (44.9%) and HFpEF (44.9%). Hypertensive changes were most common in HFpEF (63.4%) and HFmrEF (57.6%), while atrial fibrillation prevalence was highest in HFpEF (48.2%).

Echocardiographic findings (Figure 2B): Grade III mitral regurgitation and severe pulmonary hypertension each affected 25.4% of HFmrEF patients, while in HFrEF grade III MR (28.6%) and severe PH (28.6%) predominated. HFpEF was characterized by grade II MR (42.9%) and moderate PH (42.9%).

Hemoglobin concentrations (Figure 2C): Mean hemoglobin was lowest in HFmrEF (8.39 ± 1.79 g/dL), intermediate in HFrEF (8.62 ± 1.94 g/dL), and highest in HFpEF (9.07 ± 2.47 g/dL). A dashed line marks the WHO anemia threshold, showing that all mean values fell below this cut-off.

These patterns highlight phenotype-specific variation in both cardiac structure/function and hematologic profile, supporting the need for tailored diagnostic and management strategies.

### 3.5. Anemia Management During Hospitalization

Anemia management strategies during hospitalization reflected clinical severity, availability of iron parameters, and phenotype-specific treatment responses. Four primary therapeutic approaches were identified: oral iron supplementation, intravenous (IV) iron (ferric carboxymaltose or iron (III) hydroxide–sucrose), and blood transfusions. The treatment distribution varied markedly across HF phenotypes, aligning with differences in anemia severity, underlying etiology, and institutional protocols.

In the HFmrEF group, anemia management was aggressive and multifaceted, consistent with the high prevalence of severe anemia (54.2%). IV iron therapy was the dominant strategy, with 84.8% of patients receiving iron (III) hydroxide–sucrose and 66.1% receiving ferric carboxymaltose. Despite this, nearly 15.3% required red blood cell transfusions, underscoring the depth of hematologic compromise. Oral iron supplements were administered to fewer than half the patients. Adverse effects associated with IV iron were relatively infrequent but included nausea (9.3%), gastrointestinal intolerance (8.9%), and taste disturbances (5.6%). Oral iron was less well tolerated, with constipation occurring in 7.0% of treated patients.

In this group, intravenous iron was widely used, but in adjusted analyses there was no statistically significant association with in-hospital mortality (OR 1.42, 95% CI 0.07–28.56). Propensity-score-weighted analyses gave similar null findings (OR 2.23, 95% CI 0.00–1285), reflecting severe treatment imbalances and positivity violations. Given the observational design and the limited number of untreated HFmrEF patients, these results do not allow for causal inference.

The HFpEF cohort, while predominantly exhibiting mild-to-moderate anemia, also received intensive iron supplementation. IV iron was used in 75.0% (iron (III) hydroxide–sucrose) and 66.1% (ferric carboxymaltose) of patients, with oral iron administered in approximately 52% of cases. Blood transfusions were required in 16.1%, a rate slightly higher than in HFmrEF, possibly reflecting comorbid factors such as gastrointestinal bleeding or renal dysfunction. Notably, this group reported higher gastrointestinal intolerance to oral therapy (27.7%) and a broader spectrum of IV-related side effects, including Fishbane reaction (an acute, non-allergic infusion reaction characterized by flushing, hypotension, and chest discomfort) (5.1%), nausea (7.6%), and taste changes (5.6%).

In the HFrEF group, anemia treatment appeared to be more conservative. While 69.9% received iron (III) hydroxide–sucrose and 52.6% received ferric carboxymaltose, only 35.0% received oral iron supplementation, and transfusion was required in just 5.6% of cases—the lowest across all groups. This trend aligns with the lower incidence of severe anemia observed in this phenotype. Adverse events for IV therapy were similar to those in other groups (nausea, 10.2%; Fishbane reaction, 9.3%; headache, 5.4%), while oral iron intolerance (27.7%) and taste disturbances (20.5%) were commonly reported.

### 3.6. Comorbidities

Comorbidities likely influenced both anemia pathogenesis and treatment decisions, reinforcing the multidimensional profile of these patients, as seen in Table 1.

In the HFrEF cohort, structural cardiac disease and ischemic burden were predominant. Dilated cardiomyopathy was present in 58.7% of patients—the highest among all groups—followed closely by ischemic coronary artery disease in 52.1%. Grade II arterial hypertension (30.5%) and osteoarthritis (26.8%) were also frequently reported. Atrial arrhythmias such as long-standing persistent atrial fibrillation (15.5%) were common, along with a history of coronary artery bypass grafting (13.6%). Notably, both dyslipidemia and malignancy were observed in 13.6% of patients, while carotid stenosis was documented in 12.7%.

The HFmrEF group exhibited a distinct comorbidity profile. Ischemic coronary artery disease was observed in 44.9% of patients, and grade II hypertension in 25.0%. Chronic obliterative arteriopathy (22.0%) and paroxysmal atrial fibrillation (19.5%) were also prevalent. Dilated cardiomyopathy was diagnosed in 15.3%—substantially lower than in HFrEF—while dyslipidemia and malignancy were equally prevalent (13.8%). Hepatic impairment, likely linked to systemic congestion or chronic inflammation, was identified in 13.3% of patients.

Patients with HFpEF showed a different pattern, consistent with age-related and metabolic comorbidities. Ischemic coronary artery disease remained common (45.5%), while grade II hypertension increased in prevalence (37.5%). Dyslipidemia (36.6%) and osteoarthritis (33.9%) were notably higher in this cohort. Chronic obliterative arteriopathy was reported in 25.0%, while atrial fibrillation, primarily permanent, affected 21.4%. Prior coronary stent implantation was recorded in 21.4% of patients. Neurovascular and degenerative disorders were also represented, with Parkinson’s disease in 15.2% and carotid stenosis in 14.3%.

### 3.7. Discharge Pharmacotherapy

Discharge pharmacotherapy patterns reflected both heart failure (HF) guideline-directed medical therapy (GDMT) and individualized management of anemia and comorbidities, differing notably across the three HF phenotypes. We present in Table 2 the discharge medication breakdown by cohort.

In the HFrEF cohort, treatment aligned closely with standard recommendations. Diuretics were prescribed in 73.2% of patients, followed by angiotensin-converting enzyme (ACE) inhibitors in 54.9% and beta-blockers in 47.0%, indicative of established neurohormonal modulation. Proton-pump inhibitors were also frequently used (61.5%), possibly related to polypharmacy or gastrointestinal comorbidity. Iron supplementation was prescribed in 37.1% of cases, suggesting moderate integration of anemia-specific care. Statins (34.7%), aspirin (36.6%), and anticoagulants—particularly acenocoumarol (32.4%)—were commonly administered, reflecting concurrent vascular disease. Use of clopidogrel (21.6%), amiodarone (21.1%), and digoxin (20.2%) further underscores a tailored approach to arrhythmia and ischemic burden.

In HFmrEF, discharge regimens were similarly intensive. Diuretics (85.6%) and proton-pump inhibitors (79.7%) dominated pharmacotherapy, alongside beta-blockers (49.2%). Iron therapy—administered in 48.3% of patients—was more frequent than in other cohorts, potentially reflecting the higher prevalence of severe anemia in this group. Antiplatelet therapy included aspirin (39.8%) and clopidogrel (30.5%), with statins also prescribed in 30.5%. ACE inhibitors (24.6%) and angiotensin receptor blockers (ARBs, 25.4%) were used in nearly equal proportions. Amiodarone (24.6%), digoxin (15.3%), and anticoagulants, including dabigatran (23.7%) and acenocoumarol (21.2%), rounded out rhythm and thromboembolic prevention strategies, with lower use of apixaban (2.6%) and rivaroxaban (1.7%).

The HFpEF group displayed a more conservative pharmacologic profile, consistent with the lack of definitive GDMT for this phenotype. Diuretics remained the most commonly prescribed agent (83.9%), followed by aspirin (52.7%), iron supplementation (42.9%), and beta-blockers (42.9%). Statins (42.0%) and proton-pump inhibitors (58.0%) were also frequently used, often linked to vascular risk and polypharmacy. ARBs (32.1%) and ACE inhibitors (21.4%) were employed selectively. Anticoagulant use centered around acenocoumarol (25.0%), while digoxin (21.4%) was used in rhythm management.

### 3.8. Baseline Characteristics by Anemia Status and HF Phenotype

Baseline characteristics of HF patients with and without anemia, stratified by HF phenotype, are presented in Table 3. Across all phenotypes, anemic patients were older, more often female, and had a higher prevalence of comorbidities such as renal dysfunction, pulmonary hypertension, and valvular disease compared with non-anemic patients. NT-proBNP elevation was more common among anemic patients in all phenotypes.

### 3.9. In-Hospital Mortality

In-hospital mortality varied substantially across heart failure phenotypes (Table 4). Of the 443 patients included, 88 (19.9%) died during hospitalization. HFpEF patients exhibited the highest in-hospital mortality rate at 31.2%, followed by HFrEF at 22.1%. In contrast, the HFmrEF cohort experienced significantly lower mortality at just 5.1%. These findings suggest a potentially more favorable short-term prognosis for HFmrEF patients despite their high burden of severe anemia and comorbidities. While causality cannot be inferred from these observational data, the marked inter-phenotype differences underscore the importance of individualized risk stratification and management approaches. These findings suggest potential phenotype-specific differences in acute clinical outcomes, though statistical testing was not performed due to the descriptive nature of this analysis.

The observed differences in in-hospital mortality across phenotypes were statistically significant (*p* < 0.001, chi-square test). Post hoc pairwise comparisons revealed that HFpEF had significantly higher mortality than HFmrEF (*p* < 0.001) and HFrEF (*p* = 0.042), supporting a distinct short-term risk profile despite differences in anemia burden.

Values are adjusted odds ratios (aORs) with 95% confidence intervals (CIs) from a multivariable logistic regression model including HF phenotype and relevant clinical covariates. The reference category for HF phenotype is HFrEF.

In the multivariable logistic regression model including HF phenotype, anemia status, and key clinical covariates (Table 5), anemia remained independently associated with higher in-hospital mortality, adjusted OR = 1.42, 95% CI = 1.06–1.89, *p* = 0.018), independent of HF phenotype.

To account for potential confounding factors, a multivariable logistic regression analysis was performed including HF phenotype, age, sex, NYHA class, renal dysfunction (IRC), anemia status, NT-proBNP high, hypertension grade, coronary artery disease, atrial fibrillation, COPD, diabetes, pulmonary hypertension, any bleeding, and hemodynamic instability (Table 5). In this adjusted model, the lower mortality observed for HFmrEF in the univariate analysis was no longer statistically significant when compared with HFrEF (adjusted OR = 1.38, 95% CI = 0.84–2.27, *p* = 0.203). HFpEF remained associated with higher in-hospital mortality relative to HFrEF (adjusted OR = 1.76, 95% CI = 1.10–2.82, *p* = 0.019). Anemia status was independently associated with increased odds of mortality across phenotypes (adjusted OR = 1.42, 95% CI = 1.06–1.89, *p* = 0.018).

### 3.10. Exploratory Cumulative Readmission Analysis

**Figure 3:**
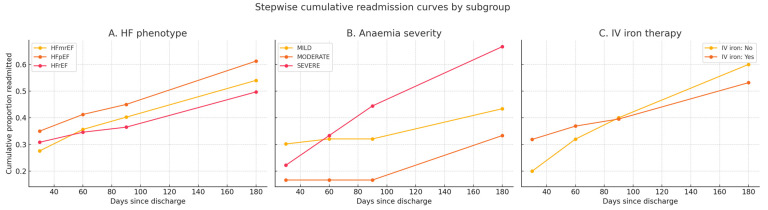
Stepwise cumulative readmission curves by HF phenotype, anemia severity, and IV iron therapy status. Panel (**A**): cumulative readmission curves for patients discharged alive, stratified by HF phenotype (HFrEF, HFmrEF, HFpEF). Panel (**B**): cumulative readmission curves stratified by anemia severity (mild, moderate, severe). Panel (**C**): cumulative readmission curves stratified by receipt of intravenous (IV) iron therapy during index hospitalization (Yes/No). Readmissions are presented as cumulative proportions within fixed post-discharge windows (≤30, ≤60, ≤90, ≤180 days). Patients who died in-hospital were excluded. This exploratory analysis shows the cumulative proportion of patients readmitted within fixed time windows after discharge, plotted for each subgroup. HFpEF patients demonstrated the highest cumulative readmission at all timepoints (Panel (**A**)), whereas readmission rose with anemia severity (Panel (**B**)). IV iron exposure was not associated with clear differences in readmission patterns (Panel (**C**)). Because follow-up dates were unavailable, these curves are descriptive and do not represent formal Kaplan–Meier survival estimates. Abbreviations: HF, heart failure; HFrEF, heart failure with reduced ejection fraction; HFmrEF, heart failure with mildly reduced ejection fraction; HFpEF, heart failure with preserved ejection fraction; IV, intravenous.

Among patients discharged alive, cumulative readmission increased across all phenotypes through 180 days. HFpEF showed the highest cumulative readmission at each time point (Figure 3), though group differences were modest (χ^2^ *p* at ≤30/≤60/≤90/≤180 days = 0.31/0.79/1.00/0.65). Readmission rose with anemia severity (from mild to severe; *p*-values at each timepoint provided in Table 6). No significant differences were observed between patients receiving versus not receiving IV iron during the index admission (*p*-values in Table 7).

These analyses are descriptive and based on fixed readmission windows (≤30, ≤60, ≤90, and ≤180 days), rather than exact event dates; as such, they should not be interpreted as formal time-to-event survival curves.

### 3.11. Subgroup Cumulative Readmission Analyses

**Figure 4:**
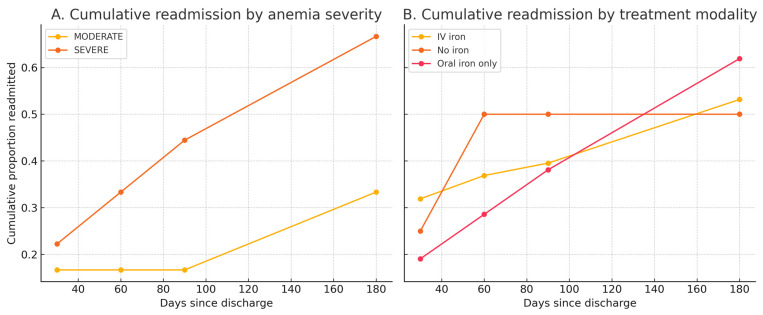
Cumulative readmission by anemia severity and treatment modality. Panel (**A**): stepwise cumulative readmission curves for patients discharged alive, stratified by anemia severity (moderate vs. severe). Panel (**B**): stepwise cumulative readmission curves for patients discharged alive, stratified by treatment modality (IV iron, oral iron only, no iron). Readmissions were assessed within fixed post-discharge intervals (≤30, ≤60, ≤90, and ≤180 days). Points represent the cumulative proportion of patients with ≥1 readmission by each timepoint; connecting lines are for visual guidance only. These curves are descriptive and are not Kaplan–Meier survival estimates. Abbreviations: IV, intravenous; MODERATE, moderate anemia; SEVERE, severe anemia.

Among patients discharged alive, cumulative readmission increased over 180 days for both moderate and severe anemia (Figure 4, Panel A). Group differences were not statistically significant at any time point (χ^2^ *p* at ≤30/≤60/≤90/≤180 days = 1.00/0.712/0.365/0.284).

Cumulative readmission curves were broadly similar across treatment modalities (IV iron, oral-only, no iron) (Figure 4, Panel B). The global χ^2^ *p*-value at ≤30/≤60/≤90/≤180 days was 0.453/0.637/0.904/0.731, indicating no significant differences. Group sizes were highly imbalanced (IV, *n* = 301; oral-only, *n* = 21; no iron, *n* = 4).

Among patients discharged alive, cumulative readmission increased over 180 days for both moderate (*n* = 12) and severe (*n* = 9) anemia (Figure 4, Panel A). Group differences were not statistically significant at any time point (the χ^2^ *p*-values at ≤30, ≤60, ≤90, and ≤180 days were 1.00, 0.712, 0.365, and 0.284, respectively).

Cumulative readmission curves were broadly similar across treatment modalities (IV iron, *n* = 301; oral iron only, *n* = 21; no iron, *n* = 4) (Figure 4, Panel B). The χ^2^ *p*-values at ≤30, ≤60, ≤90, and ≤180 days were 0.453, 0.637, 0.904, and 0.731, respectively. Interpretation of these results is limited by the marked imbalance in subgroup sizes, particularly the very small number of patients in the no-iron group.

In an adjusted logistic model of in-hospital mortality restricted to moderate and severe anemia, severe vs. moderate was not significantly associated with death (adjusted OR 1.25, bootstrap 95% CI 0.63–2.57).

### 3.12. Predictors of Heart Failure Phenotype: Multivariable Analysis

As shown in Table 8, multinomial logistic regression identified anemia severity as an independent predictor of HF phenotype. Compared with HFmrEF (the reference group), moderate anemia was significantly more likely in HFrEF patients (adjusted OR = 1.98, 95% CI: 1.14–3.43, *p* = 0.015) and showed a strong trend in HFpEF (adjusted OR = 1.63, 95% CI: 1.14–2.41, *p* = 0.009). Severe anemia also demonstrated a non-significant trend toward higher prevalence in HFrEF (adjusted OR = 2.80, 95% CI: 0.96–8.20, *p* = 0.061). Neither rural residence nor the presence of atrial fibrillation was independently associated with HF phenotype after adjustment (*p* > 0.05). These findings highlight anemia severity as a potential discriminator among HF subtypes and warrant further investigation in prospective studies.

## 4. Discussion

This descriptive analysis of 443 patients with concurrent HF and anemia provides a detailed assessment of phenotype-specific trends in clinical presentation, anemia severity, treatment modalities, and comorbid burden. Our findings underscore real-world differences between the HFrEF, HFmrEF, and HFpEF phenotypes and highlight the importance of tailoring anemia management strategies accordingly. By integrating anemia management patterns with clinical and paraclinical profiles, we extend the descriptive scope beyond previous prevalence-focused studies.

In adjusted models, anemia severity was independently associated with HF phenotype. These findings reinforce pathophysiological distinctions between phenotypes and argue for phenotype-specific screening strategies.

While intravenous iron was widely used, particularly in HFmrEF, the lack of uniform protocols and high reliance on transfusions in certain subgroups point to the need for standardized, evidence-based approaches. The HFmrEF cohort, in particular, exhibited a disproportionate burden of severe anemia and received more aggressive anemia management, including higher rates of IV iron and transfusions. Unlike prior registries, our cohort includes granular phenotyping of anemia-related symptoms, ECG/echo features, and iron therapy tolerability stratified by HF type in a real-world Eastern European population.

The inclusion of a comparator cohort of HF patients without anemia enabled us to directly examine the prognostic relevance of anemia across HF subtypes. After risk adjustment, anemia remained an independent predictor of in-hospital mortality in all phenotypes, supporting its role as a clinically important comorbidity in HF.

Our data confirm and extend the robust evidence supporting intravenous (IV) iron in HFrEF. Pivotal trials such as FAIR-HF demonstrated that ferric carboxymaltose markedly improves exercise capacity and symptoms [4], and CONFIRM-HF confirmed sustained benefits in NYHA class and quality of life over one year [12]. AFFIRM-AHF further showed that early IV iron post-decompensation reduces rehospitalizations at 52 weeks [14]. More nuanced insights emerged from the IRONMAN trial, which identified that patients with lower baseline transferrin saturation derive the greatest hemoglobin and functional improvements with ferric derisomaltose [16]. Importantly, concerns about infection risk have been largely allayed. Secondary analyses reported no increase—and possibly even a reduction—in serious infections following IV iron in HFrEF [18]. A recent Bayesian meta-analysis reinforced these findings, demonstrating a 25% relative risk reduction in HF hospitalizations with IV iron [13].

Although prior large-scale registries have examined anemia in HF populations, few have provided such detailed stratification across LVEF phenotypes in a hospitalized cohort. These data are especially relevant for health systems in underrepresented regions, such as Eastern Europe. Our findings also reaffirm the considerable symptom burden in anemic HF patients and suggest that targeted, phenotype-specific interventions may be warranted. In contrast, evidence for IV iron in HFpEF and HFmrEF remains scarce. HFpEF accounts for nearly half of all HF cases and is characterized by diastolic and endothelial dysfunction in the setting of systemic inflammation [2]. Small, single-center pilot studies have suggested that IV iron may improve exercise capacity in HFpEF [19], but these were underpowered for hard outcomes. HFmrEF, now recognized as a distinct phenotype in the 2021 ESC guidelines [2], lies between HFrEF and HFpEF pathophysiologically, yet lacks dedicated investigation. Our study highlights that HFmrEF patients receive disproportionately more transfusions and oral iron, often in the setting of subclinical renal impairment, underscoring the urgent need for targeted trials in this intermediate group.

By delineating phenotype-specific anemia profiles and management strategies, we extend the paradigm of precision cardiology to include hematologic comorbidity. A one-size-fits-all approach to iron repletion overlooks key inter-phenotype differences, such as the attenuated erythropoietic response in HFpEF despite similar hepcidin elevations, or the heightened reliance on transfusions in HFmrEF. These findings argue for tailored, cluster-specific algorithms: early IV iron in HFrEF per current guidelines [20], pilot dosing regimens in HFmrEF, and inflammation-modulating strategies in HFpEF.

### 4.1. Anemia Prevalence and Severity

Severe anemia was most prevalent in the HFmrEF cohort (54.2%), contrasting with mild anemia predominating in both HFpEF (52.1%) and HFrEF (52.1%). Earlier observational registries reported anemia rates of 20–30% in HFrEF, with severe anemia accounting for 10–15% [10,21]. The heightened incidence of severe anemia in our HFmrEF subgroup may reflect referral bias toward decompensated patients or a unique inflammatory milieu in HFmrEF, as suggested by recent biomarker analyses demonstrating altered transferrin saturation and proteomic associations in HF [22]. HFpEF patients often exhibit milder anemia, echoing community-based cohorts such as the TOPCAT trial (*N* = 1765), which linked even mild anemia to poorer outcomes in HFpEF [23].

### 4.2. Clinical Presentation

Symptoms of decompensation varied by phenotype. HFpEF patients reported dyspnoea (75%) and fatigability (58.9%) at higher rates, congruent with diastolic dysfunction and impaired preload reserve [22,24]. HFmrEF and HFrEF cohorts displayed classic volume overload signs (peripheral oedema, crepitant rales, orthopnoea) consistent with reduced systolic function. Anemia-related complaints (chest pain, anorexia, palpitations, presyncope) were frequent across all cohorts, aligning with pathophysiologic studies showing that anemia exacerbates myocardial oxygen mismatch and can precipitate angina even in non-ischemic HF [25,26].

Notably, despite a lower prevalence of severe anemia, the HFpEF cohort exhibited the highest in-hospital mortality (31.2%), a paradox that likely reflects the cumulative burden of age-related frailty, metabolic comorbidities, and impaired physiological reserve. This group demonstrated the highest prevalence of permanent atrial fibrillation, dyslipidemia, osteoarthritis, and neurodegenerative conditions, which may have contributed to vulnerability during hospitalization. Unlike HFrEF, where aggressive guideline-directed therapy and structured protocols are more common, HFpEF management remains less standardized, potentially compounding risks. These findings highlight how non-cardiac comorbidities drive short-term outcomes in HFpEF, independent of anemia severity.

#### Laboratory, Electrocardiographic, and Echocardiographic Findings

Mean hemoglobin levels differed significantly (HFmrEF, 8.39 ± 1.79 g/dL; HFpEF, 9.07 ± 2.47 g/dL; HFrEF, 8.62 ± 1.94 g/dL), suggesting more advanced anemia in the HFmrEF and HFrEF phenotypes. These values mirror recent community-based analyses in which HFrEF patients had a mean hemoglobin level of 9.0 g/dL, whereas HFpEF patients averaged 10.0 g/dL [27].

On electrocardiography, hypertensive changes predominated in HFpEF (63.4%), underscoring the critical role of hypertension in HFpEF pathogenesis [28]. Atrial fibrillation was most frequent in HFpEF (48.2%), consistent with the atrial remodeling and diastolic dysfunction documented in HFpEF [29,30,31]. Ischemic-lesion patterns were prominent in HFrEF (>50%), reflecting a higher burden of coronary artery disease, as corroborated by recent registry data [14].

Echocardiography revealed phenotype-specific distributions of valvular and pulmonary pressure abnormalities. In HFmrEF, grade III mitral regurgitation and severe pulmonary hypertension each affected 25.4% of patients, suggesting that intermediate systolic dysfunction can still provoke significant valvular insufficiency and postcapillary pulmonary hypertension [32]. HFpEF patients exhibited grade II mitral regurgitation (42.9%) and moderate pulmonary hypertension (42.9%), in line with findings from the PREFER-HF registry [23]. HFrEF patients had grade III regurgitation and severe pulmonary hypertension (28.6%), similar to IRONMAN’s HFrEF cohort, where 30% demonstrated comparable echocardiographic severity [22,33].

### 4.3. Anemia Therapy and Safety

Intravenous iron therapy—predominantly iron (III) hydroxide–sucrose—was the mainstay of anemia management. Utilization rates were high across cohorts (HFmrEF 84.8%, HFpEF 75.0%, and HFrEF 69.9%). Ferric carboxymaltose was also widely used (HFmrEF 66.1%, HFpEF 66.1%, and HFrEF 52.6%). These figures surpass those in prior randomized HFrEF trials (e.g., FAIR-HF (HFrEF 50%) [4], CONFIRM-HF (HFrEF 60%) [14]), likely reflecting evolving clinician practice patterns following publication of the IRONMAN trial [15] and contemporary ESC guidelines [34].

Blood transfusion rates were highest in HFpEF (16.1%) and HFmrEF (15.3%) compared with HFrEF (5.6%), possibly because IV iron effectively corrected anemia in HFrEF, reducing transfusion needs. Adverse events aligned with published safety data (nausea: HFmrEF 9.3%, HFpEF 7.6%, and HFrEF 10.2%; gastrointestinal intolerance: HFmrEF 8.9%, HFpEF 8.9%, and HFrEF 27.7%; Fishbane reaction: HFmrEF 5.1%, HFpEF 5.1%, and HFrEF 9.3%) [14,35]. Oral-iron-related intolerance was notably higher in HFrEF (27.7%), consistent with prior real-world cohorts [35].

Although intravenous iron was widely administered in HFmrEF, adjusted analyses did not demonstrate a statistically significant association with lower in-hospital mortality in HFmrEF or across phenotypes. Interpretation is constrained by confounding by indication, near-universal treatment in HFmrEF, and the absence of randomized allocation; therefore, causal inferences cannot be drawn.

### 4.4. Comorbidity Profiles

Comorbidities demonstrated phenotype-specific burdens. HFrEF patients had a high prevalence of dilated cardiomyopathy (58.7%) and ischemic coronary artery disease (52.1%), echoing the IRONMAN [22] and AFFIRM-AHF trial findings of 60% ischemic etiology [14]. HFmrEF exhibited a mixed profile (ischemic disease, 44.9%; chronic obstructive arteriopathy, 22.0%; paroxysmal atrial fibrillation, 19.5%), consistent with HFmrEF’s intermediate characteristics documented in the subgroup analyses of PARAGON-HF [36]. HFpEF patients carried classic metabolic syndrome comorbidities: hypertension (37.5%), dyslipidemia (36.6%), and osteoarthritis (33.9%), mirroring TOPCAT’s HFpEF cohort [23] and recent real-world studies [31,37]. Elevated rates of atrial fibrillation (21.4%) and prior stent implantation (21.4%) in HFpEF underscore overlapping pathophysiologic mechanisms linking coronary microvascular dysfunction, atrial remodeling, and diastolic HF [29,30]. Our observation of a high prevalence of anemia in HFpEF, coupled with the highest in-hospital mortality among phenotypes, aligns with prior evidence linking anemia to adverse outcomes in this subgroup. In particular, a recent study [38] demonstrated a significant association between anemia and long-term mortality specifically in patients with HFpEF, reinforcing the prognostic importance of anemia in this phenotype. Unlike our retrospective in-hospital analysis, that study evaluated long-term outcomes, underscoring the need for prospective research to determine whether targeted anemia management can improve survival in HFpEF.

### 4.5. Discharge Pharmacotherapy

At discharge, diuretics remained the most frequently prescribed therapy (HFmrEF 85.6%, HFpEF 83.9%, and HFrEF 73.2%), consistent with literature data [20,39]. Proton-pump inhibitor use was high (>58%) across cohorts, reflecting gastrointestinal prophylaxis in polypharmacy contexts. Beta-blocker and ACE inhibitor/ARB prescription rates were highest in HFrEF (46.9% and 54.9%, respectively), reflecting landmark trials such as PARADIGM-HF [40] and DAPA-HF3 and existing data [41,42]. In HFpEF, neurohormonal antagonist use was lower (beta-blockers 42.9%, ACE inhibitors 21.4%), consistent with evidence that such therapies improve outcomes only in HFpEF subpopulations with specific phenotypes [41]. Antiplatelet (aspirin, clopidogrel) and anticoagulant (acenocoumarol, dabigatran) therapies were most prevalent in HFmrEF, aligning with high ischemic burden and atrial fibrillation rates. Continued iron supplementation at discharge (HFmrEF 48.3%, HFpEF 42.9%, and HFrEF 37.1%) indicates sustained recognition of iron deficiency as a modifiable risk factor.

### 4.6. Clinical Implications and Future Directions

Our findings provide descriptive insights that could inform the design of future prospective studies aimed at improving outcome-based care for anemia in HF. While exploratory, our data point to phenotype-specific disparities in anemia management that merit further investigation in randomized trials. We emphasize that any management implications discussed are hypothesis-generating and not prescriptive, given the retrospective and non-interventional design of our study. We acknowledge that the identification of predictors of anemia severity from baseline characteristics would be of great clinical interest. However, due to the retrospective nature of the data, heterogeneity in clinical assessments, and lack of uniform documentation, multivariable modeling was not performed in this analysis.

In-hospital mortality data, though limited, revealed a notably lower event rate in HFmrEF compared with the HFrEF and HFpEF phenotypes. In the adjusted logistic regression model (Table 5), the previously observed lower mortality for HFmrEF did not remain statistically significant (adjusted OR = 1.38, 95% CI = 0.84–2.27, *p* = 0.203), suggesting that differences in age, functional status, renal function, and comorbidity burden may account for much of the crude mortality variation. In contrast, HFpEF retained an independent association with higher in-hospital mortality (adjusted OR = 1.76, 95% CI = 1.10–2.82, *p* = 0.019), and anemia itself was an independent predictor of mortality across HF phenotypes (adjusted OR = 1.42, 95% CI = 1.06–1.89, *p* = 0.018). These findings highlight the importance of risk adjustment when comparing outcomes between phenotypes and reinforce the need for prospective studies to confirm phenotype-specific prognostic patterns. The notably lower mortality in HFmrEF compared with HFrEF and HFpEF supports its proposed intermediate risk profile, although prospective validation is needed.

While our analysis was primarily descriptive, we recognize the importance of providing greater statistical transparency. In particular, the presentation of confidence intervals and *p*-values in the Results section can be refined to better contextualize the strength and precision of the associations observed. For example, the adjusted odds ratio for severe anemia predicting HFrEF versus HFmrEF was 2.80 (95% CI: 0.96–8.20, *p* = 0.061), suggesting a clinically relevant trend that did not reach conventional statistical significance. This near-significant finding may indicate a true underlying association that could be confirmed in larger, powered studies. Therefore, readers should interpret these estimates as hypothesis-generating rather than conclusive. Future prospective analyses with larger sample sizes and longer follow-up periods are warranted to validate these preliminary associations.

Strengths of our analysis include its large, unselected patient population and comprehensive phenotypic characterization. However, its retrospective design, single-center setting, and lack of long-term follow-up limit causal inference. Residual confounding, particularly around iron dosing decisions and clinician preference, cannot be excluded. Future prospective RCTs are needed to validate our observations, especially in HFmrEF and HFpEF, and to explore mechanistic biomarkers that may refine patient selection. Such trials will be essential to optimize anemia management, minimize rehospitalizations, and move toward truly personalized care across the heart failure spectrum.

Our study further underscores the critical value of recognizing HF phenotypes as distinct clinical clusters that demand bespoke therapeutic strategies. By delineating the nuanced anemia profiles and treatment responses in HFrEF, HFmrEF, and HFpEF cohorts, we extend the paradigm of precision cardiology beyond ejection-fraction cut-offs to encompass hematologic comorbidity. Our study emphasizes the need for phenotype-tailored anemia strategies in heart failure. HFrEF patients showed a relatively milder anemia profile with lower transfusion rates and greater adherence to guideline-directed therapies. In contrast, HFmrEF exhibited the highest burden of severe anemia and IV iron use, while HFpEF patients—despite having more moderate anemia—experienced the highest in-hospital mortality, likely due to multimorbidity and frailty. These findings reinforce the importance of recognizing the distinct clinical and therapeutic patterns among HF phenotypes when designing future interventions. These insights also advocate for phenotype-tailored iron repletion algorithms (e.g., early IV iron in HFrEF, pilot dosing regimens in HFmrEF, and inflammation-modulating strategies in HFpEF) and pave the way for cluster-specific clinical trials. Ultimately, our findings reinforce that integrating hematologic and hemodynamic phenotyping will be vital to optimize outcomes, minimize adverse events, and truly personalize [43] care across the heart failure spectrum.

Although not directly tested in our cohort, prior trials suggest potential synergy between SGLT2 inhibitors and IV iron. Our findings support further investigation of such combinations in prospective studies. Indeed, the success of SGLT2 inhibitors across the ejection-fraction spectrum in trials such as DAPA-HF (hazard ratio (HR) = 0.74; 95% CI: 0.65–0.85) [3] and EMPEROR-Preserved (HR = 0.83; 95% CI: 0.71–0.98) [44] underscores the feasibility of broad-based, phenotype-stratified interventions. Moreover, meta-analytic data demonstrate that SGLT2 inhibition reduces the risk of cardiovascular death or HF hospitalization by 23% regardless of LVEF [45], raising the possibility of synergistic benefits when combined with iron repletion. Although not based on cohort-specific data, prior trials suggest that SGLT2 inhibitors and IV iron may have synergistic effects. Our findings offer a rationale for such combinations to be tested in future prospective studies.

Future randomized trials should therefore incorporate dual-pathway designs testing IV iron plus SGLT2 inhibition in HFmrEF and HFpEF, stratify by biomarkers [46] such as hepcidin and NT-proBNP to identify responders, and assess hard outcomes including HF hospitalizations, cardiovascular mortality, and patient-reported quality of life. Finally, implementation science efforts will be critical to translate these insights into practice, ensuring that targeted anemia management becomes an integral component of precision HF care. All therapeutic implications discussed are exploratory in nature and should not be construed as clinical recommendations.

## 5. Limitations

This study is subject to several limitations, including its retrospective design, single-center setting, and lack of follow-up data. Consequently, causal inferences and outcome predictions cannot be made. Moreover, no multivariable models were employed for long-term outcomes or for predictors of anemia severity; treatment decisions were left to the clinician’s discretion. The findings are thus intended to be descriptive and hypothesis-generating only. Importantly, all patients included in this study had anemia by WHO criteria; thus, comparisons with non-anemic heart failure patients were not feasible and are beyond the scope of this work. The lack of post-discharge clinical outcomes precludes assessment of prognostic implications, which should be explored in future longitudinal or interventional studies. Although in-hospital mortality was assessed, no follow-up data were available to evaluate long-term prognosis, functional outcomes, or rehospitalization. The retrospective nature of our study introduces inherent selection bias. In addition, the absence of standardized institutional treatment criteria for iron supplementation and the lack of systematic documentation of the rationale for choosing intravenous versus oral iron therapy limit our ability to control for treatment selection bias. Consequently, our findings regarding iron therapy should be interpreted as observational associations rather than causal effects. Prospective studies with protocolized iron replacement strategies and standardized documentation of decision-making processes are needed to confirm and extend these results.

Due to incomplete inflammation or renal indices, full multivariate models of anemia severity predictors could not be applied.

The HFmrEF subgroup exhibited severe non-overlap in treatment propensity, with only six untreated patients, leading to unstable effect estimates despite adjustment. These limitations preclude causal interpretation of the observed associations.

Finally, follow-up dates for readmission and death were not available, precluding formal Kaplan–Meier analysis. Our cumulative readmission curves are based on fixed intervals and should be interpreted as descriptive only.

The absence of exact follow-up dates precluded formal Kaplan–Meier and log-rank analyses. Stepwise cumulative readmission curves are provided instead and should be interpreted as descriptive. Due to the small size of some subgroups (particularly the no-iron group) and the absence of exact follow-up dates, the subgroup analyses are exploratory and based on fixed-interval data and, therefore, should be interpreted with caution.

Data completeness was variable. Not all patients had full iron panels or uniform echocardiographic evaluations, introducing possible misclassification. Clinical variables were extracted from routine documentation, which may be subject to inconsistencies or omissions.

Potential confounders, such as renal function dynamics, inflammation markers, and socioeconomic status, were not consistently recorded, limiting risk adjustment. Additionally, anemia and iron therapy were managed at the treating physician’s discretion, without standardized protocols.

No longitudinal data on post-discharge outcomes (e.g., mortality, readmission, functional recovery) were available, precluding any prognostic assessment. The study also lacks validation in external populations, which would be necessary to confirm the reproducibility and generalizability of the findings.

## 6. Conclusions

This real-world, retrospective analysis describes meaningful differences in anemia severity, management practices, and comorbidity patterns across the HFrEF, HFmrEF, and HFpEF phenotypes. While not designed to assess long-term outcomes, the findings highlight key phenotype-specific trends, including the paradoxically high in-hospital mortality in HFpEF despite the lower burden of severe anemia—likely reflecting age-related frailty and multimorbidity. These results underscore the importance of incorporating HF phenotype into future studies and clinical pathways to optimize anemia management. The therapeutic insights presented are exploratory and intended to inform the design of future prospective, randomized trials aimed at determining whether targeted anemia correction can improve clinical outcomes across the heart failure spectrum.

## Figures and Tables

**Table 1:** Detailed comorbidity percentages for each HF phenotype.

Comorbidity	HFrEF (%)	HFmrEF (%)	HFpEF (%)	*p*-Value
Dilated Cardiomyopathy	58.69	15.25	-	0.00
Ischemic Coronary Artery Disease	52.11	44.92	45.54	0.44
Arterial Hypertension	30.52	25.00	37.50	0.03
Osteoarthritis	26.76	-	33.93	0.42
Chronic Obliterative Arteriopathy	-	22.03	25.00	0.12
Dyslipidemia	13.62	13.76	36.61	0.05
Malignancy	12.68	13.76	-	0.19
Carotid Stenosis	12.68	-	14.29	0.08
Long-standing Persistent Atrial Fibrillation	15.49	-	-	0.09
Paroxysmal Atrial Fibrillation	-	19.49	-	0.08
Permanent Atrial Fibrillation	-	-	21.43	0.07
Coronary Artery Bypass Grafting	13.62	-	-	0.06
Prior Stent Implantation	-	-	21.43	0.05
Hepatic Impairment	-	13.30	-	0.10
Parkinson’s Disease	-	-	15.18	0.21

HFmrEF, heart failure with mildly reduced ejection fraction; HFpEF, heart failure with preserved ejection fraction; HFrEF, heart failure with reduced ejection fraction.

**Table 2:** Discharge medication breakdown by heart failure phenotype.

Medication	HFrEF (%)	HFmrEF (%)	HFpEF (%)	*p*-Value
Diuretics	73.24	85.59	83.93	0.01
MRA	87.39	69.53	72.11	0.01
ARBs	27.94	25.42	32.14	0.01
ACE Inhibitors	54.93	24.58	21.43	0.02
Beta-blockers	46.95	49.15	42.86	0.62
Neprilysin inhibitor	72.12	69.79	58.46	0.02
SGL2i	83.76	77.93	75.42	0.03
Iron Supplements	37.09	48.31	42.86	0.02
Aspirin	36.62	39.83	52.68	0.18
Statins	34.74	30.51	41.96	0.01
Acenocoumarol	32.39	21.19	25.00	0.02
Clopidogrel	21.60	30.51	-	0.43
Amiodarone	21.13	24.58	-	0.94
Digoxin	20.19	15.25	21.43	0.08
Proton-pump Inhibitors	61.50	79.66	58.04	0.31
Dabigatran	-	23.73	-	0.09
Apixaban	-	2.64	-	0.08
Rivaroxaban	-	1.69	-	0.09

ACE, angiotensin-converting enzyme; ARB, angiotensin II receptor blockers; HFmrEF, heart failure with mildly reduced ejection fraction; HFpEF, heart failure with preserved ejection fraction; HFrEF, heart failure with reduced ejection fraction; MRA, mineralocorticoid receptor antagonist; SGL2i, sodium-glucose-cotransporter 2 inhibitors.

**Table 3:** Baseline characteristics of heart failure (HF) patients with and without anemia, stratified by HF phenotype.

HF_Phenotype	Variable	No Anemia	Anemia	*p*_Value
HFmrEF	Age (years)	76.18 ± 11.33	71.52 ± 11.35	0.03
	Female (%)	55.26	42.86	0.20
	Urban residence (%)	50.00	57.14	0.46
	NT-proBNP high (%)	73.68	71.43	0.79
	Aortic stenosis grade (mean ± SD)	0.08 ± 0.27	0.29 ± 0.71	0.20
	Coronary artery disease (%)	61.84	14.29	0.00
	Dilated cardiomyopathy (%)	7.89	28.57	0.00
	COPD (%)	22.37	14.29	0.29
	Diabetes (%)	23.68	42.86	0.03
	Renal dysfunction (IRC) (%)	23.68	14.29	0.22
	Stroke/TIA (%)	7.89	14.29	0.27
	Peripheral vascular disease (%)	15.79	14.29	0.83
	Obesity (%)	14.47	28.57	0.06
	Dyslipidemia (%)	15.79	42.86	0.00
	Any bleeding (%)	15.79	28.57	0.10
HFrEF	Age (years)	75.28 ± 9.98	74.73 ± 11.20	0.58
	Female (%)	53.16	61.82	0.27
	Urban residence (%)	56.96	61.82	0.53
	NT-proBNP high (%)	70.89	67.27	0.61
	Aortic stenosis grade (mean ± SD)	0.14 ± 0.43	0.16 ± 0.54	0.81
	Coronary artery disease (%)	58.86	32.73	0.00
	Dilated cardiomyopathy (%)	48.10	89.09	0.00
	COPD (%)	45.57	38.18	0.34
	Diabetes (%)	36.71	43.64	0.36
	Renal dysfunction (IRC) (%)	28.48	70.91	0.00
	Stroke/TIA (%)	10.13	0.00	0.01
	Peripheral vascular disease (%)	7.59	0.00	0.04
	Obesity (%)	10.13	20.00	0.06
	Dyslipidemia (%)	17.09	0.00	0.00
	Any bleeding (%)	3.80	0.00	0.14
	Hemodynamic instability (%)	3.80	0.00	0.14
HFpEF	Age (years)	74.47 ± 9.90	80.00 ± 5.48	0.14
	Female (%)	59.43	66.67	0.73
	Urban residence (%)	58.49	33.33	0.23
	NT-proBNP high (%)	65.09	50.00	0.45
	Aortic stenosis grade (mean ± SD)	0.06 ± 0.23	0.00 ± 0.00	0.55
	Coronary artery disease (%)	45.28	100.00	0.01
	COPD (%)	28.30	100.00	0.00
	Diabetes (%)	22.64	0.00	0.19
	Renal dysfunction (IRC) (%)	45.28	0.00	0.03
	Stroke/TIA (%)	21.70	100.00	0.00
	Peripheral vascular disease (%)	10.38	0.00	0.41
	Obesity (%)	11.32	0.00	0.38

Legend: Continuous variables are expressed as mean ± SD; categorical variables as percentages (%). The *p*-values reflect comparisons between HF patients with and without anemia within the same HF phenotype. “Anemia” includes mild, moderate, and severe categories according to WHO criteria; “No anemia” refers to patients without anemia by WHO criteria. Variables without any recorded cases in at least one comparator group within a phenotype are shown with 0.00%. Abbreviations: HF, heart failure; HFrEF, heart failure with reduced ejection fraction; HFmrEF, heart failure with mildly reduced ejection fraction; HFpEF, heart failure with preserved ejection fraction; NT-proBNP, N-terminal pro–B-type natriuretic peptide; COPD, chronic obstructive pulmonary disease; IRC, renal dysfunction; SD, standard deviation. Values are presented as mean ± standard deviation (SD) for continuous variables and as percentages for categorical variables. Comparisons between anemia and no-anemia groups were performed within each HF phenotype using the Chi-square test for categorical variables and the Kruskal–Wallis test for continuous variables. Only variables with at least one recorded case in both comparator groups within a phenotype are presented.

**Table 4:** In-hospital mortality by HF phenotype.

HF_Phenotype	Total Patients	In-Hospital Deaths	Mortality Rate (%)
HFrEF	213	47	22.1
HFmrEF	118	6	5.1
HFpEF	112	35	31.2

Rates are stratified by ejection fraction group among 443 anemic HF patients. Abbreviations: HF, heart failure; HFrEF, heart failure with reduced ejection fraction; HFmrEF, heart failure with mildly reduced ejection fraction; HFpEF, heart failure with preserved ejection fraction.

**Table 5:** Multivariable logistic regression for predictors of in-hospital mortality in patients with heart failure (HF).

Variable	aOR	CI	*p*_Value
Intercept	0.04	0.01–0.11	0.000
HFmrEF vs. HFrEF	1.38	0.84–2.27	0.203
HFpEF vs. HFrEF	1.76	1.10–2.82	0.019
Age (per year)	1.02	1.01–1.04	0.001
Female	1.17	0.88–1.56	0.279
NYHA III–IV	1.30	0.95–1.79	0.105
Renal dysfunction (IRC)	0.92	0.67–1.26	0.593
Anemia (any)	1.40	0.84–2.33	0.192
NT-proBNP high	1.03	0.76–1.41	0.835
Hypertension grade	1.29	1.10–1.52	0.002
Coronary artery disease	0.99	0.74–1.33	0.960
Atrial fibrillation (any)	0.87	0.61–1.22	0.407
COPD	0.87	0.64–1.18	0.355
Diabetes	1.14	0.83–1.57	0.424
Any bleeding	0.73	0.47–1.14	0.165
Hemodynamic instability	2.53	0.43–14.86	0.304

Adjusted for HF phenotype, age, female sex, NYHA III–IV, renal dysfunction (IRC), anemia (any), NT-proBNP high, hypertension grade, coronary artery disease, atrial fibrillation (any), COPD, diabetes, pulmonary hypertension, any bleeding, and hemodynamic instability. An aOR < 1 indicates lower odds of mortality compared with the reference; an aOR > 1 indicates higher odds. The *p*-values < 0.05 are considered statistically significant. Abbreviations: HFrEF, heart failure with reduced ejection fraction; HFmrEF, heart failure with mildly reduced ejection fraction; HFpEF, heart failure with preserved ejection fraction; NYHA, New York Heart Association; NT-proBNP, N-terminal pro–B-type natriuretic peptide; COPD, chronic obstructive pulmonary disease; IRC, renal dysfunction; aOR, adjusted odds ratio; CI, confidence interval.

**Table 6:** Anemia severity and cumulative readmission.

Timepoint (Days)	*p*-Value	Mild Anemia (%)	Moderate Anemia (%)	Severe Anemia (%)
30	0.60	30.19	16.67	22.22
60	0.56	32.08	16.67	33.33
90	0.38	32.08	16.67	44.44
180	0.30	43.40	33.33	66.67

Cumulative readmission by anemia severity. Stepwise readmission proportions demonstrate a progressive increase in readmission risk with greater anemia severity. These findings are descriptive and based on fixed readmission intervals (≤30, ≤60, ≤90, ≤180 days), not exact follow-up times. Cumulative proportions of patients readmitted within fixed post-discharge windows, stratified by anemia severity (mild, moderate, severe). Only patients discharged alive were included. Readmissions are reported as a percentage of patients in each group at each time point. Group comparisons were performed using chi-square tests; *p*-values are reported for each time point. Abbreviations: MILD, mild anemia; MODERATE, moderate anemia; SEVERE, severe anemia.

**Table 7:** Iron therapy and cumulative readmission.

Timepoint (Days)	*p*-Value	IV Iron: No (%)	IV Iron: Yes (%)
30	0.31	20	31.89
60	0.79	32	36.88
90	1.00	40	39.53
180	0.65	60	53.16

Cumulative readmission by IV iron therapy. Curves and percentages show similar readmission patterns for patients with and without IV iron exposure during index hospitalization. Analyses are descriptive, based on fixed readmission intervals (≤30, ≤60, ≤90, ≤180 days), and should not be interpreted as formal time-to-event survival curves. Cumulative proportions of patients readmitted within fixed post-discharge windows, stratified by receipt of intravenous (IV) iron during the index hospitalization. Only patients discharged alive were included. Readmissions are reported as a percentage of patients in each group at each time point. Group comparisons were performed using chi-square tests; *p*-values are reported for each time point. Abbreviations: IV, intravenous.

**Table 8:** Multivariable predictors of HF phenotype.

Phenotype	Predictor	Adjusted OR	95% CI Lower	95% CI Upper	Std. Error	*p*-Value
HFpEF	Atrial Fibrillation (Any)	1.4	0.81	2.41	0.28	0.22
HFpEF	Rural Residence	0.84	0.5	1.42	0.27	0.51
HFrEF	Moderate Anemia	1.98	1.14	3.43	0.28	**0.015**
HFrEF	Severe Anemia	2.8	0.96	8.2	0.55	0.061

Multinomial logistic regression model identifying independent predictors of HF phenotype. The reference category for phenotype comparison is HFmrEF. Odds ratios (ORs) represent the adjusted likelihood of each predictor being associated with HFpEF or HFrEF versus HFmrEF. *p*-values are derived from multinomial logistic regression. Values in bold denote statistical significance at *p* < 0.05. Abbreviations: HFrEF, heart failure with reduced ejection fraction; HFpEF, heart failure with preserved ejection fraction; OR, odds ratio; CI, confidence interval; Std. Error, standard error.

## Data Availability

The datasets analyzed in the current study are available from the corresponding author on reasonable request.

## Abbreviations

Abbreviation	Definition
ACE	Angiotensin-Converting Enzyme
AF	Atrial Fibrillation
ANOVA	Analysis of Variance
ARB	Angiotensin Receptor Blocker
CBC	Complete Blood Count
CI	Confidence Interval
CKD	Chronic Kidney Disease
ECG	Electrocardiography
ESC	European Society of Cardiology
FDA	U.S. Food and Drug Administration
HF	Heart Failure
HFrEF	Heart Failure with Reduced Ejection Fraction
HFmrEF	Heart Failure with Mid-Range Ejection Fraction
HFpEF	Heart Failure with Preserved Ejection Fraction
HR	Hazard Ratio
ICD	Implantable Cardioverter-Defibrillator
IV	Intravenous
LVEF	Left Ventricular Ejection Fraction
NYHA	New York Heart Association
NT-proBNP	N-terminal pro–B-type Natriuretic Peptide
OR	Odds Ratio
RAAS	Renin–Angiotensin–Aldosterone System
RCT	Randomized Controlled Trial
SD	Standard Deviation
SGLT2	Sodium–Glucose Cotransporter 2
WHO	World Health Organization
